# Supplementary material for: GC-MS Analysis and Biomedical Therapy of Oil from n-Hexane Fraction of Scutellaria edelbergii Rech. f.: In Vitro, In Vivo, and In Silico Approach
Source: Molecules. 2021 Dec 18;26(24):7676. doi: 10.3390/molecules26247676 (PMC8706644; doi:10.3390/molecules26247676)
Supplement: Supplementary file 1 [file molecules-26-07676-s001.zip › molecules-1496530-SI.pdf]

molecules

Supporting information

Article

GC-MS Analysis and Biomedical Therapy of Oil from n-Hexane Fraction of *Scutellaria edelbergii Rech*. f.: In Vitro, In Vivo, and In Silico Approach

Muddaser Shah [1,2], Waheed Murad [1,*], Najeeb Ur Rehman [2,*], Sidra Mubin [3], Jamal Nasser Al-Sabahi [4], Manzoor Ahmad [5], Muhammad Zahoor [6], Obaid Ullah [2,5], Muhammad Waqas [2,7], Saeed Ullah [2,8], Zul Kamal [9,10], Rafa Almeer [11], Simona G. Bungau [12] and Ahmed Al-Harrasi [2,*]

[1] Department of Botany, Abdul Wali Khan University Mardan, Mardan 23200, Pakistan; muddasershah@awkum.edu.pk
[2] Natural and Medical Sciences Research Center, University of Nizwa, P.O. Box 33, Birkat Al Mauz, Nizwa 616, Oman; obaidullah@unizwa.edu.om (O.U.); mwaqas@unizwa.edu.om (M.W.); saeedullah@iccs.edu (S.U.)
[3] Department of Botany, Hazara University Mansehra, Mansehra 21310, Pakistan; shahhu123@gmail.com
[4] Central Instrument Laboratory, College of Agriculture and Marine Sciences, Sultan Qaboos University, Muscat 123, Oman; jamal@squ.edu.om
[5] Department of Chemistry, University of Malakand, Chakdara 18800, Pakistan; manzoorhej@yahoo.com
[6] Department of Biochemistry, University of Malakand, Chakdara 18800, Pakistan; mohammadzahoorus@yahoo.com
[7] Department of Biotechnology and Genetic Engineering, Hazara University, Mansehra 21120, Pakistan
[8] H.E.J. Research Institute of Chemistry, International Center for Chemical and Biological Science, University of Karachi, Karachi 75270, Pakistan
[9] Department of Pharmacy, Shaheed Benazir Bhutto University, Upper Dir 18000, Pakistan; xulkamal@sbbu.edu.pk
[10] School of Pharmacy, Shanghai Jiao Tong University, Minhang 800, Shanghai 200240, China
[11] Department of Zoology, College of Science, King Saud University, P.O. Box 2455, Riyadh 11451, Saudi Arabia; ralmeer@ksu.edu.sa
[12] Department of Pharmacy, Faculty of Medicine and Pharmacy, University of Oradea, Oradea 410028, Romania; simonabungau@gmail.com
* Correspondence: waheedmurad@awkum.edu.pk (W.M.); najeeb@unizwa.edu.om (N.U.R.); aharrasi@unizwa.edu.om (A.A.-H.)

molecules

Table S1. Chemical structures of the compounds detected through GC-MS analysis in the crude oils isolated from n-hexane fraction of *S. edelbergii.*

S. No	Compound	Chemical structures	MW (g/mol)
1	Thymol	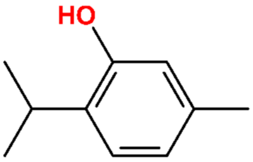	150.22
2	1-Tridecene	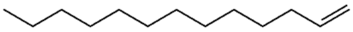	182.35
3	2,4-Di-tert-butylphenol	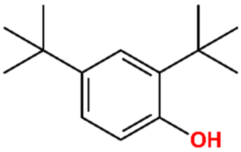	206.32
4	Cetene	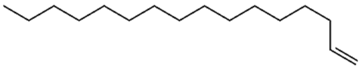	224.42
5	Dodecanoic acid, ethyl ester	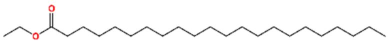	228.37
6	Hexadecanoic acid, methyl ester	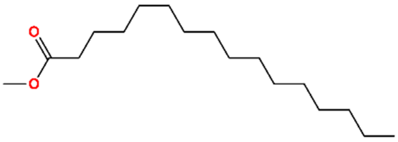	270.5
7	Methyl tetradecanoate	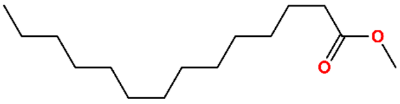	242.4
8	α-Octadecene	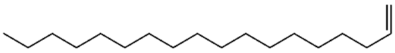	252.5
9	Myristic acid, ethyl ester	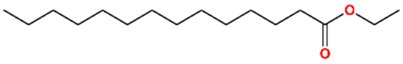	256.42
10	Phytol, acetate	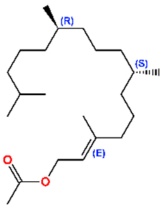	338.6
11	Isopropyl myristate	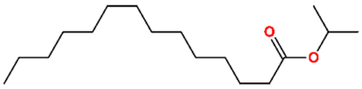	270.5
12	Hexahydrofarnesyl acetone	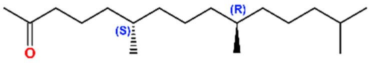	268.5
13	Pentadecanoic acid, ethyl ester	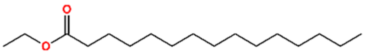	270.5

molecules MDPI

14	Methyl hexadec-9-enoate	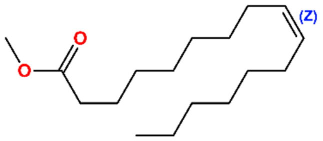	268.4
15	Hexadecanoic acid, methyl ester	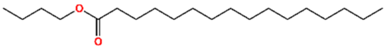	270.5
16	Ethyl 9-hexadecenoate	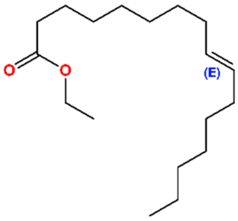	282.5
17	Palmitic acid, ethyl ester	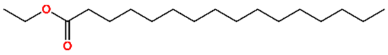	284.5
18	Isopropyl palmitate	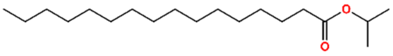	298.5
19	Heptadecanoic acid, methyl ester	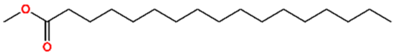	284.5
20	Linoleic acid, methyl ester	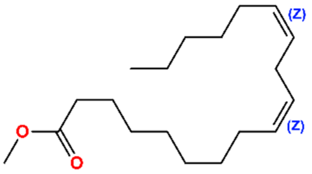	294.5
21	Linolenic acid, methyl ester	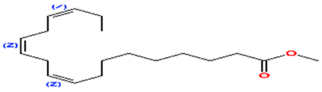	292.5
22	Oleic acid, methyl ester	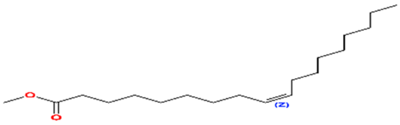	296.5
23	Methyl stearate	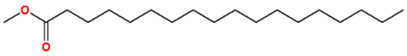	298.5
24	Dodecyl nonyl ether	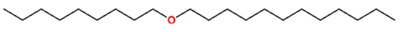	312.6
25	Linoleic acid ethyl ester	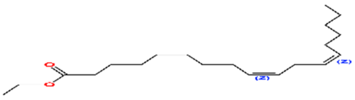	308.5
26	Ethyl Oleate	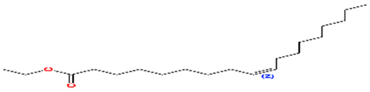	310.5
27	Hexadecanoic acid, butyl ester	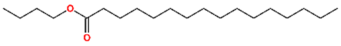	312.5
28	Octadecanoic acid, ethyl ester	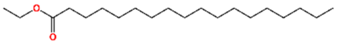	312.5

molecules

MDPI

29	Heneicosane		296.6
30	cis-11-Eicosenoic acid, methyl ester	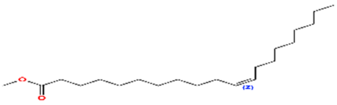	324.5
31	Eicosanoic acid, methyl ester	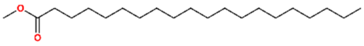	326.6
32	13-Docosenoic acid, methyl ester, (Z)-	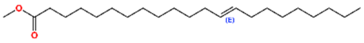	352.6
33	Docosanoic acid, methyl ester	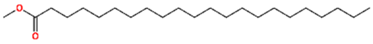	354.6
34	Docosanoic acid, ethyl ester	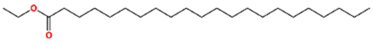	368.6
35	Tricosanoic acid, methyl ester	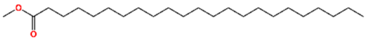	368.6
36	2-Methylhexacosane	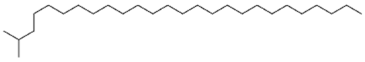	380.7
37	15-Tetracosenoic acid, methyl ester, (Z)-	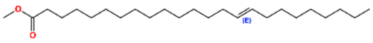	380.6
38	Tetracosanoic acid, methyl ester	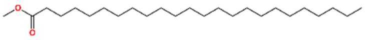	382.7
39	(E)-3,7-Dimethylocta-2,6-dien-1-yl palmitate	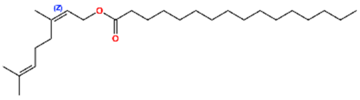	392.7
40	Squalene	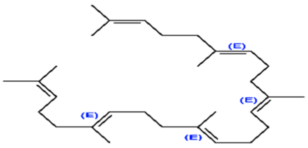	410.7
41	1,6,10,14,18,22-Tetracosahexaen-3-ol, 2,6,10,15,19,23-hexamethyl-, (all-E)-	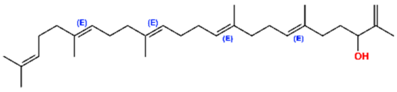	426.7
42	Tetracontane	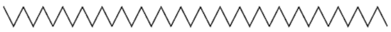	563.1
